# Network-level permutation entropy of resting-state MEG recordings: A novel biomarker for early-stage Alzheimer’s disease?

**DOI:** 10.1162/netn_a_00224

**Published:** 2022-06-01

**Authors:** Elliz P. Scheijbeler, Anne M. van Nifterick, Cornelis J. Stam, Arjan Hillebrand, Alida A. Gouw, Willem de Haan

**Affiliations:** Alzheimer Center Amsterdam, Department of Neurology, Amsterdam Neuroscience, Vrije Universiteit Amsterdam, Amsterdam, Netherlands; Department of Clinical Neurophysiology and MEG Center, Department of Neurology, Amsterdam Neuroscience, Vrije Universiteit Amsterdam, Amsterdam, Netherlands

**Keywords:** Biomarker, Functional brain networks, Joint permutation entropy, Early-stage Alzheimer’s, Magnetoencephalography

## Abstract

Increasing evidence suggests that measures of signal variability and complexity could present promising biomarkers for Alzheimer’s disease (AD). Earlier studies have however been limited to the characterization of local activity. Here, we investigate whether a network version of permutation entropy could serve as a novel biomarker for early-stage AD. Resting-state source-space magnetoencephalography was recorded in 18 subjects with subjective cognitive decline (SCD) and 18 subjects with mild cognitive impairment (MCI). Local activity was characterized by permutation entropy (PE). Network-level interactions were studied using the inverted joint permutation entropy (JPE_inv_), corrected for volume conduction. The JPE_inv_ showed a reduction of nonlinear connectivity in MCI subjects in the theta and alpha band. Local PE showed increased theta band entropy. Between-group differences were widespread across brain regions. Receiver operating characteristic (ROC) analysis of classification of MCI versus SCD subjects revealed that a logistic regression model trained on JPE_inv_ features (78.4% [62.5–93.3%]) slightly outperformed PE (76.9% [60.3–93.4%]) and relative theta power–based models (76.9% [60.4–93.3%]). Classification performance of theta JPE_inv_ was at least as good as the relative theta power benchmark. The JPE_inv_ is therefore a potential biomarker for early-stage AD that should be explored in larger studies.

## Author Summary

Functional network disruption is a well-established finding in Alzheimer’s disease. Sensitive network-based biomarkers are however not available. We aimed to detect neuronal dysfunction at a predementia (mild cognitive impairment, MCI) stage of Alzheimer’s disease, by applying a network-level neural variability measure to magnetoencephalography data: the inverted joint permutation entropy (JPE_inv_). This measure integrates information on local signal variability/complexity and nonlinear coupling. We found significant differences in JPE_inv_ between subjects with subjective cognitive decline and MCI, primarily in the theta band. The diagnostic ability of the JPE_inv_ was reported to be similar to that of relative theta power, the most potent neurophysiological biomarker of predementia Alzheimer’s disease to date.

## INTRODUCTION

In the past decade, complex network approaches have increasingly been used to comprehend the structure and function of brain networks, in healthy subjects as well as in patients with neurological and psychiatric disorders (Bullmore & Sporns, 2009; Douw et al., 2019; Stam, 2014; van den Heuvel et al., 2019). Alzheimer’s disease (AD) is the major cause of dementia in the aging Western population and has been a principal target for network studies (Babiloni et al., 2020; Pievani et al., 2011). Abnormalities of structural and functional networks have been demonstrated in AD, including its earliest stages (Pusil et al., 2019; M. Yu et al., 2021). Initially, the focus was on the loss of the “small-world” organization that is seen in healthy subjects, and the shift towards more random network topology (Stam et al., 2009; Stam et al., 2007; Supekar et al., 2008). More recent studies have emphasized the selective vulnerability of highly connected hub regions (M. Yu et al., 2017). Complementary to clinical studies, large-scale computational network models of AD have provided new analytical opportunities and insights, for instance by suggesting the concept of “activity-dependent degeneration” as a cause of synaptic failure and hub vulnerability, or by relating amyloid-driven neuronal excitation/inhibition imbalance to the well-known large-scale oscillatory slowing in AD (de Haan et al., 2012; Maestú et al., 2021; Stefanovski et al., 2021; Stefanovski et al., 2019). Insights derived from such network-oriented models can be used to develop and test potential new treatments in a simulated environment, and thereby guide future clinical studies (de Haan et al., 2017).

While network approaches have helped to gain a better understanding of mechanisms involved in the pathophysiology of AD, it is not clear whether measures derived from network analysis could also serve as effective biomarkers—especially in the early stages of AD. Graph theoretical analysis of brain networks is complex and has been hampered by methodological problems. Reconstruction of functional networks from resting-state recordings of functional magnetic resonance imaging (fMRI), electroencephalography (EEG), or magnetoencephalography (MEG), for instance, raises questions about the proper use of thresholds, error due to movement, volume conduction, and state changes such as drowsiness and sleep (van Diessen et al., 2015; van Wijk et al., 2010). These methodological issues may be responsible for the limited reproducibility and reliability of connectivity and network measures, although recent studies do suggest improvement in this respect (Briels et al., 2020; Colclough et al., 2016). In addition to robustness and good reproducibility, clinically useful biomarkers need sensitivity and specificity of at least 80% or higher (Babiloni et al., 2021; Colom-Cadena et al., 2020). Thus far, mainly conventional, single-channel power spectral measures have proven their diagnostic and predictive value in the predementia stages of AD (Gouw et al., 2017; Gouw et al., 2021; Horvath et al., 2018; Hughes et al., 2019). While it is still entirely conceivable that the richness of brain network dynamics can help to detect or distinguish early aberrant neuronal behavior, the question arises whether progress can be made with a different type of approach, that somehow combines the best of both worlds.

Several research groups have emphasized the importance of studying the variability or complexity of neural dynamics, at different spatial and temporal scales (Garrett et al., 2013; McIntosh et al., 2010; Uddin, 2020; Waschke et al., 2021). A central idea is that a certain level of variability in neural activity corresponds to a healthy state with flexible responses to internal and external stimuli (Courtney & Hinault, 2021; Garrett et al., 2013; Waschke et al., 2021; D. Yin & Kaiser, 2021). Neural variability has been shown to relate to age as well as cognitive and behavioral performance (Angulo-Ruiz et al., 2021; Boylan et al., 2021; Dustman et al., 1999; Gómez et al., 2013; Kumral et al., 2020). Neural variability may also reflect the activity of modulating noradrenergic, dopaminergic, and cholinergic systems, and has been related to the excitation/inhibition balance in neural networks (Bruining et al., 2020; Gao et al., 2017; Garrett et al., 2015; Maestú et al., 2021; Pfeffer et al., 2021; Wang et al., 2018; Zheng et al., 2012). It could therefore in theory relate AD pathophysiology at the cell/circuit level, such as the amyloid-induced hyperexcitability mentioned above, to oscillatory changes at the larger scale—a highly desired translational quality to develop a multiscale mechanistic description of AD neurophysiology.

A wide range of measures can be used to quantify variability of time series of neural activity. Of special interest is a large group of measures that are based on the concept of information entropy (Bandt & Pompe, 2002; Costa et al., 2002, 2005; Courtiol et al., 2016; Dávalos et al., 2019; Fadlallah et al., 2013; Inouye et al., 1991; Keshmiri, 2020; Kosciessa et al., 2020; Richman & Moorman, 2000; Y. Yin et al., 2016). Entropy measures have been helpful in psychophysiological studies of healthy subjects (Fekete et al., 2021; Grady & Garrett, 2018; M. Liu et al., 2019; Mahjoory et al., 2019; Miskovic et al., 2019; Waschke et al., 2019), and growing evidence suggests that the measures can also be used to demonstrate a significant, progressive loss of entropy of neural activity in AD patients (Ando et al., 2021; Echegoyen et al., 2020; Gómez & Hornero, 2010; Maturana-Candelas et al., 2019; Shumbayawonda et al., 2020; Su et al., 2021; W. Y. Yu et al., 2021). This suggests that measures of complexity or entropy could have potential as biomarkers for AD. A limitation of previous studies is that they only consider local activity and do not take into account interregional network communication, which is known to be affected in AD (Babiloni et al., 2020; Engels et al., 2015; Engels et al., 2017; Pievani et al., 2011). Recently developed measures apply concepts of variability or information entropy to relations between multiple signals (Baracchini et al., 2021; Godfrey & Singh, 2021; Jamin & Humeau-Heurtier, 2020; King et al., 2013; Lee et al., 2017; L. Liu et al., 2010). One example is the joint permutation entropy (JPE), which makes it possible to analyze local complexity and interregional nonlinear coupling in a single comprehensive analysis (Y. Yin et al., 2019).

In the present study, we investigated whether the inverted JPE (JPE_inv_) is a potential biomarker for early AD. The JPE_inv_ was applied to resting-state source-space MEG recordings of 18 subjects with subjective cognitive decline and 18 subjects with predementia AD. The measure was computed in relevant frequency bands and was modified to prevent effects of volume conduction/field spread (King et al. 2013). The magnitude of JPE_inv_ group differences was compared with a relative theta power benchmark and with local complexity findings, in order to determine the added value of the network-level measure of entropy. The discriminative power of theta JPE_inv_, local permutation entropy, and relative power were evaluated using logistic regression models.

## MATERIALS AND METHODS

### Subjects

The study involved two age- and gender-matched groups totaling 36 subjects: 18 subjects with subjective cognitive decline (SCD) and 18 subjects with amnestic mild cognitive impairment (MCI). Data were obtained from the Amsterdam Dementia Cohort (van der Flier & Scheltens, 2018). All subjects visited the memory clinic of the VUmc Alzheimer Center in the period of spring 2015–18 and provided written informed consent for the use of their data for research purposes. Each subject received a standardized diagnostic workup including medical history taking, neurological and neuropsychological examination, blood tests, 3T MRI of the brain, routine MEG, and, when possible, a lumbar puncture to collect cerebrospinal fluid (van der Flier & Scheltens, 2018). Diagnoses were generated during a multidisciplinary consensus meeting according to the 2011 National Institute on Aging–Alzheimer’s Association (NIA-AA) criteria. Positive amyloid biomarkers (cerebrospinal fluid ptau/amyloid ratio > 0.020 and/or abnormal amyloid PET) were available for all 18 (amnestic) MCI subjects. The SCD group included 15 amyloid-negative subjects and three subjects with unknown biomarker status. Demographic characteristics of the included subjects are presented in Table 1. Average Mini–Mental State Examination (MMSE) scores were significantly lower in MCI than in SCD subjects (*p* < 0.01). Psychoactive medication use (not shown here) was incidental and did not differ significantly between groups.

**Table 1.** Demographic characteristics of the included subjects. MCI = Mild cognitive impairment. M/F = male/female. MMSE = Mini–Mental State Examination. SCD = subjective cognitive decline. *SD* = standard deviation. ** *p* < 0.01 (MCI versus SCD).

	SCD	MCI
*n*	18	18
Age in years (mean ± *SD*)	64.2 ± 6.1	64.1 ± 6.2
M/F (*n*)	8/10	9/9
MMSE (mean ± *SD*)	27.8 ± 2.1	25.8 ± 1.9**

### MEG Recordings

MEG recordings were obtained in a magnetically shielded room using a 306-channel whole-head Vectorview MEG system (Elekta Neuromag Oy, Helsinki, Finland). The acquisition protocol consisted of at least two 5-min blocks of eyes-closed recording. Subjects were instructed to relax but stay awake. Only data from the first eyes-closed session were analyzed here. Recordings were sampled at 1,250 Hz with an online anti-aliasing filter (410 Hz) and high-pass filter (0.1 Hz). A 3D-digitizer (Fastrak, Polhelmus, Colchester, VT, USA) was used to digitize the locations of four or five head position indicator coils, which were used to continuously record the subjects’ head position in relation to the MEG sensors. To provide an outline of the subjects’ scalp, ∼500 additional points were digitized. The scalp surface was used for coregistration with the structural (MRI) template that produced the best fit.

### MEG Source Reconstruction

The temporal extension of the signal space separation (tSSS) filter (implemented in MaxFilter software, Elekta Neuromag Oy, version 2.2.15; Taulu & Simola, 2006) was used to suppress correlated noise. Channels that contained excessive artefacts (i.e., flat, very noisy and squid-jump channels) were discarded based on visual inspection of the raw data, before estimation of the tSSS coefficients. The (denoised) signal was then reconstructed for all sensors (Taulu et al., 2004; Taulu et al., 2005). In order to obtain source-localized activity for all regions, an atlas-based beamforming approach was applied (Hillebrand et al., 2012). The broadband MEG data (0.5–70 Hz) were projected through the beamformer spatial filters in order to reconstruct time series of neuronal activity for 78 cortical regions of interest (ROIs) plus both hippocampi, identified by means of automated anatomical labeling (AAL; Gong et al., 2009; Tzourio-Mazoyer et al., 2002; Supplementary Table 1 in the Supporting Information). The centroid voxel of each AAL region was used as representative for that ROI (Hillebrand et al., 2016). The sphere that best fitted the scalp surface obtained from the coregistered MRI scan was used as a volume conductor model. The volume conductor model, an equivalent current dipole, and the MEG data covariance matrix were used to compute the broadband beamformer weights. By projecting sensor-level MEG data through the normalized beamformer weights (Cheyne et al., 2007), time series of neuronal activity were obtained for each ROI.

### Time Series Analysis

The source-reconstructed time series were converted to ASCII format. The first 20 epochs of 4,096 samples (3.2768 s) of the first eyes-closed recording were selected for analysis. Quantitative spectral as well as local and network-level entropy analyses were performed using in-house developed software (BrainWave, version 0.9.163.26, available from home.kpn.nl/stam7883/brainwave.html). The epochs were filtered in canonical frequency bands, i.e., theta (4–8 Hz), alpha (8–13 Hz), beta (13–30 Hz), and broadband (0.5–45 Hz) using a discrete fast Fourier transform. Relative theta power, permutation entropy, and inverted joint permutation entropy measures were estimated for each epoch separately and averaged per person prior to group statistics.

### Permutation Entropy and Inverted Joint Permutation Entropy

Computation of the permutation entropy (PE) was based on the work by Bandt and Pompe (2002). Consider a discrete time series *X*(*t* = 1, …, *T*). In the present study, this time series corresponds to the local activity of a brain region, as reconstructed by an MEG beamformer. For each time point *t* in the time series we can construct a vector with length n: (*x*_*t*+1_, …, *x*_*t*+*n*_). Next, *n* rank values are assigned to each sample within the vector, such that the sample with the highest amplitude gets rank 1, the sample with the second-highest amplitude gets rank 2, and so on to the sample with the lowest value, which gets rank *n*. There are *n*! different possible permutations of a set of *n* ranks. Each different permutation can be considered a unique symbol that can be designated with a letter. Physiologically, each letter reflects a sequence of *n* data points of an MEG time series, recorded from a single brain region. The time series *X*_*t*_ has now been converted to a sequence of n! different symbols. The probability for the occurrence of a permutation is defined by the following:

$$p\left(\pi \right)=\frac{\#\left\{t|t\leq T-n,\;\left(x_{t+1},\ldots ,x_{t+n}\right)has\;\text{type}\;\pi \right\}}{T-n+1}.$$ (1)

From the probabilities we can construct a probability distribution with *n*! bins. The Shannon information entropy of this distribution is given by the following:

$$H\left(n\right)=-\sum p\left(\pi \right)\log p\left(\pi \right).$$ (2)

The maximum value of the entropy is log(*n*!). A normalized version of the permutation entropy can be obtained as follows: PE(*n*) = *H*(*n*)/log(*n*!). Bandt and Pompe (2002) have shown that the permutation entropy does not depend strongly upon the choice of *n*. They recommend choosing *n* in the range 3–7, so that *n*! << *T*.

The PE is a robust measure of the complexity of a single time series. Recently, an extension to multivariate time series, referred to as the joint permutation entropy (JPE), was proposed by Y. Yin et al. (2019). In this approach, a symbolic representation for each of two time series is obtained as described above. Next, a matrix is constructed where each cell contains the probability of one of the (*n*! × *n*!)^2^ combinations of symbols in the two channels. This matrix forms a probability distribution with (*n*! × *n*!)^2^ bins. From this, the JPE can be computed according to Formula (2). In this study, the JPE was normalized between 0 and 1 by dividing it by its maximum value, that is, log(*n*! × *n*! − 2*n*!)^2^. The normalization factor reflects the number of bins in the probability distribution matrix that was used to compute the JPE. Signal spread can cause spurious correlations, also in the case of source-reconstructed MEG data (Hillebrand et al., 2012). To address this problem, all pairs of identical or mirrored symbols (i.e., the diagonals of the probability distribution matrix; 2*n*! bins) were excluded from computation of the JPE. This approach is similar to the suggestion by King et al. (2013).

Intuitively, we would expect a measure of nonlinear coupling to have higher values if coupling is stronger. This is, by definition of the term, not true for the JPE. In order to facilitate comparison to conventional connectivity measures, we introduce the *inverted* JPE, so that higher JPE_inv_ values correspond to stronger coupling:

$${JPE}_{inv}=1-JPE.$$ (3)

**Figure 1.**
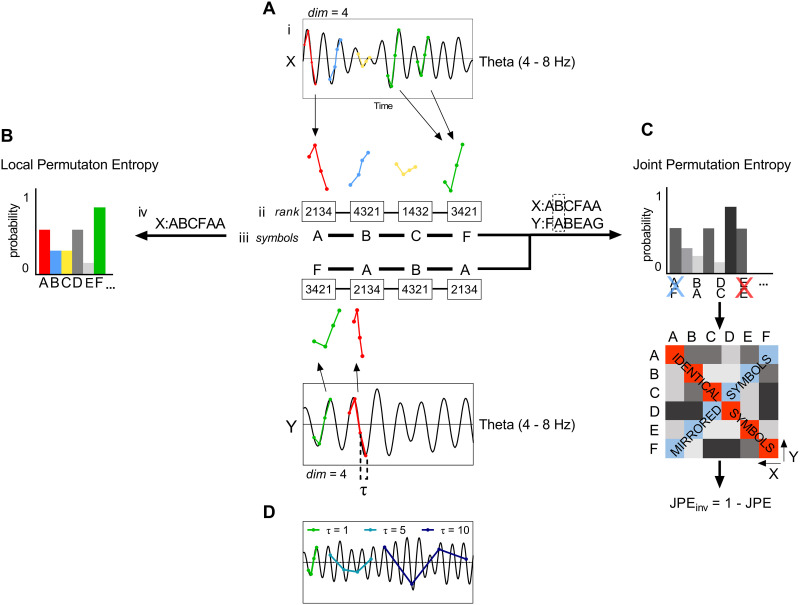
Illustration of local permutation entropy and inverted joint permutation entropy analysis of multichannel recordings of brain activity. (A) A single-channel recording of brain activity (e.g., MEG signal) is filtered in a frequency band of interest (i). Each sample in a specified time window is assigned a rank (ii). This set of ranks, or ordinal pattern, is encoded as a symbol; in this case a letter (iii). A sequence of symbols is obtained by repeating Step ii and iii for the whole time series (iv). For a length or embedding dimension *n*, there are *n*! possible different patterns that can be assigned a unique symbol. Here, we make use of *n* = 4. (B) The frequency of occurrence of each symbol is used to obtain a probability distribution. By computing the Shannon information entropy of this distribution, we obtain the local permutation entropy (PE), allowing us to assess the complexity of a single time series. (C) A symbolic representation of a second time series is obtained as described in panel A. The frequency of occurrence of each symbol pair is used to construct a probability distribution, and a symmetric matrix that reflects the probability of occurrence of each possible symbol pair. To correct for the effect of volume conduction/field spread, symbol pairs made up of identical or mirrored ordinal patterns (i.e., the diagonals of the matrix) are excluded from analysis (King et al., 2013). The joint permutation entropy (JPE) is derived from the probabilities of the remaining symbol pairs. The measure is normalized between 0 and 1 by dividing it by its maximum value, that is, log(*n*! × *n*! − 2*n*!). Finally, the inverted JPE (JPE_inv_) is obtained. (D) Lag or time delay τ describes the relation between *n* samples of a time series, each separated by τ − 1 samples. Here, we made use of τ = 1. Larger time delays will result in permutations for increasingly slower trends in the signal.

**Figure 2.**
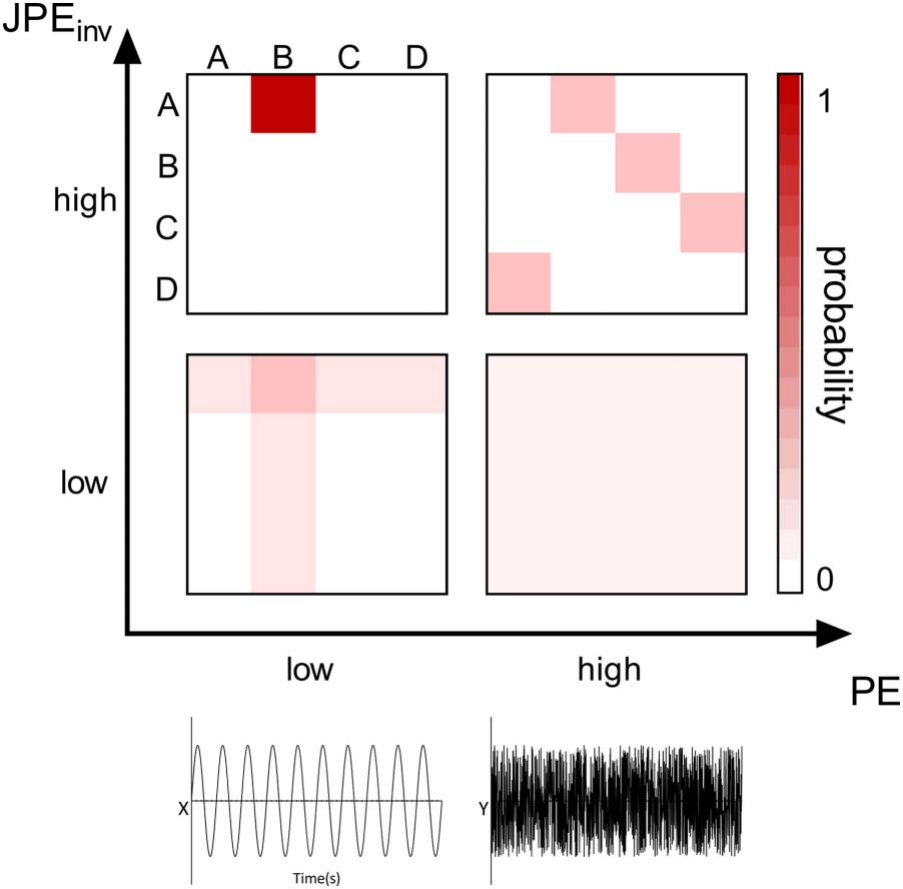
The JPE_inv_ integrates information on local complexity and interregional coupling. Let us imagine two time series, x and y, that can display varying values of PE and JPE_inv_. The symbol pair probability distributions (derived from the time series as in Figure 1C) of four possible regimes are plotted as a function of local complexity/variability (PE) and interregional coupling strength (JPE_inv_). Top left (high JPE_inv_/low PE): When both time series have low PE values, a few distinct ordinal patterns—and their corresponding symbols (e.g., A and B)—make up most of the signal. If the time series are strongly interconnected, symbol A in time series x will always be coupled to symbol B in time series y and vice versa. Bottom left (low JPE_inv_/low PE): If simple (low entropy) signals are weakly coupled, this results in coupling between symbol A in time series x and symbol B, as well as less prevalent symbols C and D, in time series y. The same holds true for symbol B in time series y: The symbol will be coupled to symbol A, as well as to less prevalent symbols C and D, in time series x. Top right (high JPE_inv_/high PE): When both time series have high PE values, each symbol (e.g., A, B, C, and D) has an equal probability of occurrence in the signal. If the time series are strongly interconnected, this results in a high probability of occurrence for specific symbol pairs (e.g., x = D & y = A, x = A & y = B, x = B & y = C, x = C & y = D). Bottom right (low JPE_inv_/high PE): If the complex (high entropy) time series are weakly coupled, this results in a low probability of occurrence of each symbol pair and a highly random signal.

A schematic illustration of the method to obtain PE and JPE_inv_ values is shown in Figure 1. Figure 2 provides intuition on how the JPE_inv_ integrates information on local complexity and interregional coupling. In the study of Y. Yin et al. (2019), the authors considered a coarse-graining procedure to obtain entropy values for different timescales. However, how coarse-graining affects the frequency content of the shortened signal is not obvious (Kosciessa et al., 2020). To avoid this problem, and to relate our results to previous work, we applied different band pass filters to the data and computed PE and JPE_inv_ for the broadband or narrowband filtered data.

### Relative Theta Power

Relative theta power is the most potent neurophysiological biomarker of predementia AD to date. The spectral measure is known to correlate with neuropsychological measures and total tau, and has been related to clinical progression over time (Gouw et al., 2017; Musaeus et al., 2018). Classifiers trained on spectral M/EEG features have achieved moderate to high accuracy rates for the classification of (early) AD and healthy control subjects. Gouw et al. (2021), for instance, obtained an accuracy rate of 84.6% for the classification of AD and SCD subjects using a random forest model—a finding that was replicated in an independent test set. The discriminative power and magnitude of group differences obtained using the proposed metrics were therefore compared to conventional, single-channel power. The potential confounding effect of oscillatory slowing on the JPE_inv_ analysis was evaluated.

### Statistical Analysis

Statistical analyses to compare the demographic characteristics of the diagnostic groups were performed in SPSS for Mac (Version 25.0. IBM Corp, Armonk, NY). Two-tailed independent sample *t* tests were performed to test the equality of group means.

Nonparametric permutation tests were performed in order to compare JPE_inv_, PE, and spectral measures between SCD and MCI subjects. The data were used to generate a probability distribution for testing against the null hypothesis, rather than that a particular distribution was assumed. The original configuration of subjects was randomly repartitioned (number of iterations = 10,000), and the permutation *p* value represented the proportion of random partitions that had a larger test statistic than the observed one. A *q* value < 0.05 (i.e., *p* value after false discovery rate correction; Benjamini & Hochberg, 1995) was considered significant.

Logistic regression models with diagnosis (SCD-MCI) as dependent variable and JPE_inv_, PE, or relative power features in the theta band as independent variables were used to assess the diagnostic value of the MEG markers. Feature values were averaged over all 80 AAL regions (i.e., 78 cortical regions and both hippocampi) before inclusion in the logistic regression models. Receiver operating characteristic (ROC) curves were plotted to quantify between-subject discrimination accuracy. Integrated area under the ROC curve (AUC) values were reported with 95% confidence intervals. In order to evaluate the potential effect of oscillatory slowing on the relationship between mean theta JPE_inv_ and diagnostic group, one-way ANCOVA with mean relative theta power as a covariate was performed.

## RESULTS

### JPE_inv_

**Figure 3.**
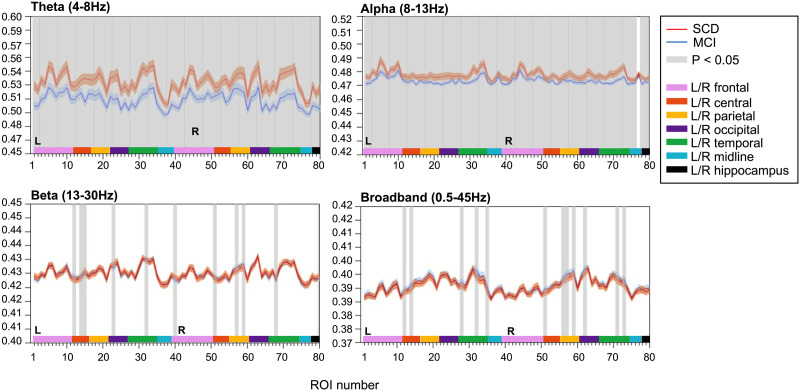
Inverted joint permutation entropy (τ = 1, *n* = 4). JPE_inv_ values were calculated for narrowband, that is, theta (4–8 Hz), alpha (8–13 Hz), beta (13–30 Hz), and broadband (0.5–45 Hz) filtered MEG data. Each ROI number (1–80, order based upon Gong et al., 2009, Supplementary Table 1) represents a brain region in the AAL atlas. Group means (±2 × SEM) are plotted in red for the SCD and in blue for the MCI group. ROIs with significantly different JPE_inv_ values (*q* < 0.05, FDR corrected) are presented in gray. The MCI group presented lower mean JPE_inv_ values for 80 regions in the theta band and for 79 channels in the alpha band. Only a few regions showed significant between-group differences in the beta (i.e., 10) and broadband (i.e., 12) data. AAL = automated anatomical labeling. FDR = false discovery rate. JPE_inv_ = inverted joint permutation entropy. MCI = mild cognitive impairment. SCD = subjective cognitive decline. SEM = standard error of the mean.

JPE_inv_ analysis (τ = 1, *n* = 4) revealed disturbed functional network coupling in MCI. Average JPE_inv_ values were significantly lower in MCI than in SCD subjects in the theta and alpha band (Figure 3). This was true for all cortical regions and both hippocampi, except for the right insula in the alpha band. Each ROI number (1–80, order based upon Gong et al., 2009, Supplementary Table 1) represents a brain region. Group differences were less distinct in beta and broadband data, with only a few cortical regions (i.e., 10–12 regions) showing statistically significant group differences in JPE_inv_-based functional connectivity (*q* < 0.05, FDR corrected). JPE_inv_ results obtained using different parameter (τ and *n*) settings are presented in the Supporting Information (Supplementary Figures 3 and 4).

### PE

**Figure 4.**
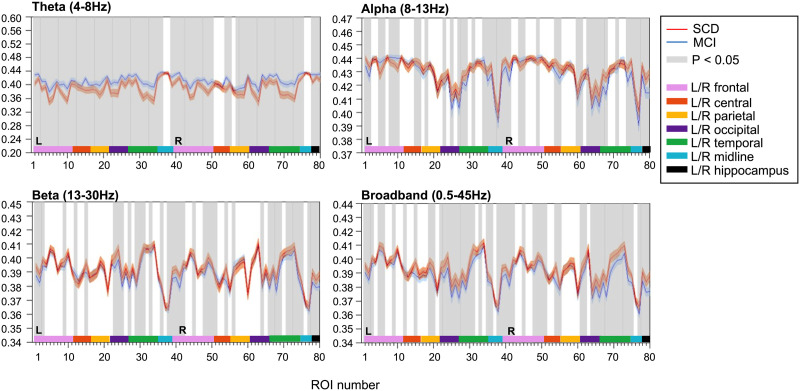
Local permutation entropy (τ = 1, *n* = 4). PE values were calculated for narrowband, that is, theta (4–8 Hz), alpha (8–13 Hz), beta (13–30 Hz), and broadband (0.5–45 Hz) filtered MEG data. Each ROI number (1–80, order based upon Gong et al., 2009, Supplementary Table 1) represents a brain region in the AAL atlas. Group means (±2 × SEM) are plotted in red for the SCD and in blue for the MCI group. ROIs with significantly different PE values (*q* < 0.05, FDR corrected) are presented in gray. Group differences were most distinct in the theta band, with 72 channels showing significantly higher PE values for MCI than for SCD subjects. AAL = automated anatomical labeling. FDR = false discovery rate. PE = permutation entropy. MCI = mild cognitive impairment. SCD = subjective cognitive decline. SEM = standard error of the mean.

Local PE analysis (τ = 1, *n* = 4) revealed differences in local activity and complexity between SCD and MCI subjects (Figure 4). Higher PE values were reported for MCI subjects in the theta band, with increased signal complexity in 70 cortical regions and both hippocampi. Although restricted to fewer regions of the brain, MCI subjects presented lower PE values than did SCD subjects in the alpha, beta, and broadband data, suggesting reduced complexity in these frequency bands. PE analysis was repeated for different values of τ and *n* (Supporting Information, Supplementary Figures 1, 2, and 5). The role of these parameters in entropy computations is addressed in the Discussion section.

### Relative Theta Power

**Figure 5.**
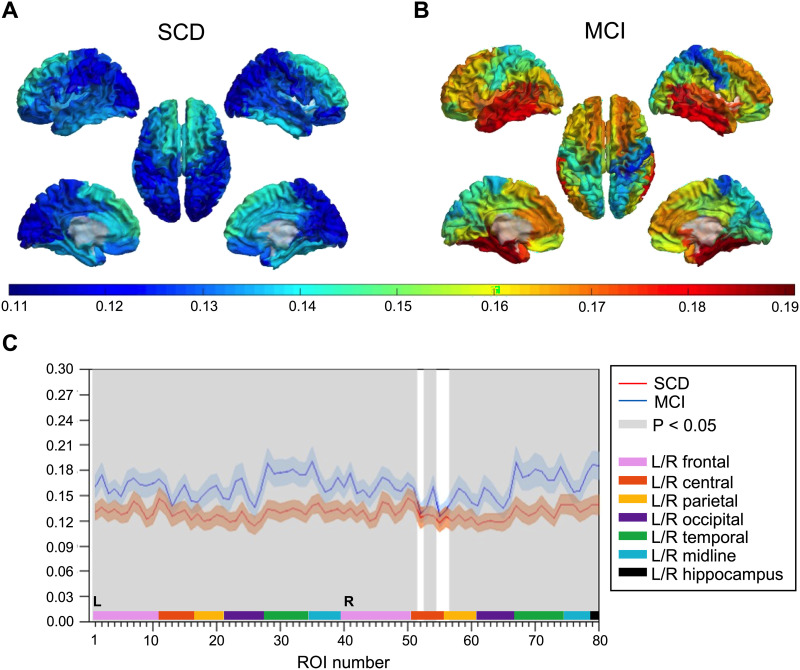
Relative theta power. (A–B) Group-averaged relative theta power values for 78 cortical brain regions are displayed as color-coded maps on a template brain, viewed from, in clockwise order, the left, top, right, right midline, and left midline. Hippocampi are not visualized. Higher relative theta power is depicted in warmer and lower power in colder colors. (A) Average relative theta power in the SCD group. (B) Average relative theta power in the MCI group. (C) Statistical analysis by permutation testing revealed higher relative theta power in MCI than in SCD subjects. Each ROI number (1–80, order based upon Gong et al., 2009, Supplementary Table 1) represents a brain region in the AAL atlas. Group means (±2 × SEM) are plotted in red for the SCD and in blue for the MCI group. ROIs with significantly different theta power values (*q* < 0.05, FDR corrected) are presented in gray. AAL = automated anatomical labeling. FDR = false discovery rate. MCI = mild cognitive impairment. SCD = subjective cognitive decline. SEM = standard error of the mean.

Spectral power analysis of local MEG activity revealed higher relative theta power in MCI than in SCD subjects (Figure 5). Seventy-five cortical regions and both hippocampi showed statistically significant group differences (*q* < 0.05, FDR corrected), with most prominent differences in parietal and temporal regions.

### Classification: ROC Analysis

**Figure 6.**
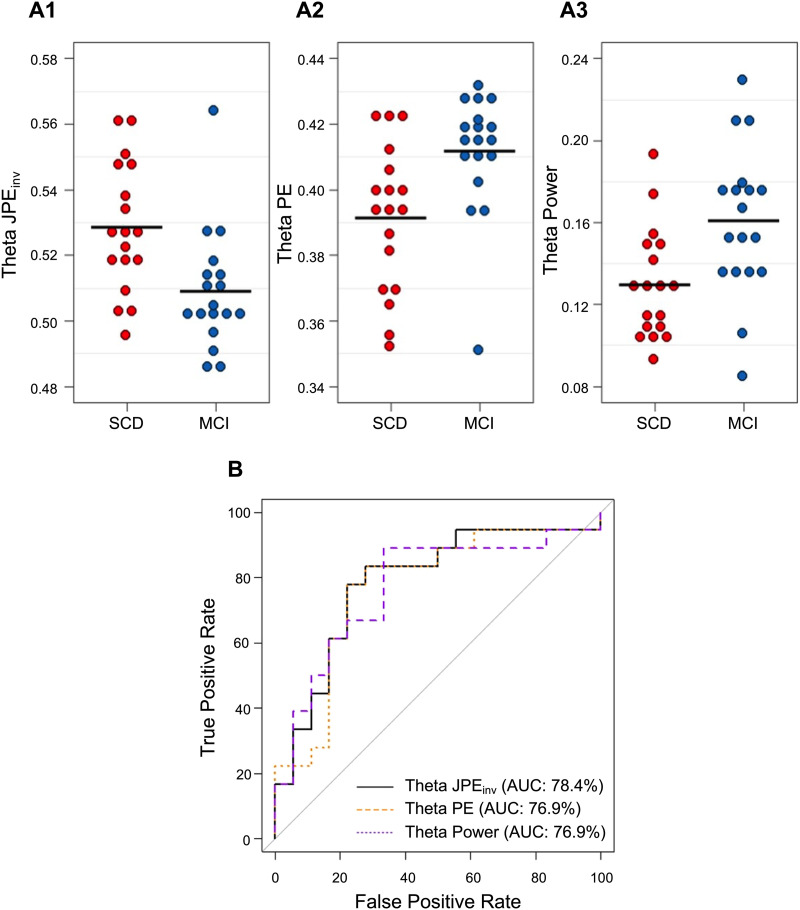
Classification of SCD and MCI subjects based on global theta JPE_inv_, PE, and relative power. (A1–A3) Each dot depicts the whole-brain average for a single subject. Black horizontal lines reflect the group means. JPE_inv_, PE, and relative power values are shown for the theta band (4–8 Hz). Visual inspection revealed that MCI subjects (depicted in blue) generally had lower JPE_inv_, higher PE, and higher relative theta power values than SCD subjects (depicted in red). (B) ROC curve analysis indicated that the highest diagnostic accuracy was achieved by the JPE_inv_-based logistic regression model (AUC = 78.4%), followed by models trained on PE and relative theta power values (AUC = 76.9%). AUC = area under the curve. JPE_inv_ = inverted joint permutation entropy. PE = permutation entropy. MCI = mild cognitive impairment. SCD = subjective cognitive decline.

As shown in previous sections, JPE_inv_ and PE group differences were most pronounced in the theta band. Average theta JPE_inv_, PE, and relative power values (i.e., averaged over all 80 ROIs) for the individual subjects are plotted in Figure 6A1–A3. Visual inspection of the data revealed that the range of the MEG metrics partially overlapped between groups. The diagnostic value of global theta JPE_inv_, PE, and relative power was therefore assessed with logistic regression models. AUCs (and 95% confidence intervals) for SCD-MCI classification per MEG metric are listed in Table 2. ROC curves for the individual predictors are plotted in Figure 6B. Highest accuracy for differentiating between SCD and MCI subjects was achieved by the JPE_inv_-based model (AUC = 78.4%), followed by the PE and relative theta power models (AUC = 76.9%). One-way ANCOVA indicated that there was a significant effect of diagnostic group on mean theta JPE_inv_ after controlling for mean relative theta power (F(1, 33) = 5.27, *p* < 0.05, η^2^ = 0.14), suggesting that the entropy variance between groups can at least partially be explained by nonlinear characteristics.

**Table 2.** Classification of SCD and MCI subjects based on theta band JPE_inv_, PE, and relative power. CI = confidence interval. JPE_inv_ = inverted joint permutation entropy. PE = permutation entropy.

MEG marker	AUC (95% CI)
Theta JPE_inv_	78.4% (62.5–93.3%)
Theta PE	76.9% (60.3–93.4%)
Relative theta power	76.9% (60.4–93.3%)

## DISCUSSION

The aim of the present study was to explore the potential of a network version of permutation entropy (JPE_inv_) as a biomarker for early-stage AD. Substantially lower JPE_inv_ values were reported in MCI than in SCD subjects in the theta and alpha frequency bands, signifying weaker network coupling in this predementia phase of AD. Local PE and relative power were higher in MCI subjects in the theta band. The biomarker potential of all three measures was evaluated using logistic regression models. The JPE_inv_-based model achieved the highest accuracy when discriminating between subjects with SCD and MCI.

### Lower Theta and Alpha JPE_inv_ in MCI

The JPE_inv_ showed significant differences between SCD and MCI subjects in the theta and alpha band, in nearly all investigated brain regions (Figure 3). It is important to realize that the joint entropy depends on both local entropy and the level of nonlinear coupling between activity of different regions (Figure 2). Low levels of noninverted JPE typically reflect a combination of low local entropy and strong interregional coupling, while high levels of noninverted JPE reflect the opposite pattern. To facilitate comparison with conventional functional connectivity measures, we introduced the inverted JPE (JPE_inv_). The lower JPE_inv_ values reported here reflect stronger local entropy in MCI (which is in line with the reported PE results) as well as lower functional connectivity. Previous studies have reported a loss of functional connectivity in AD, especially in the alpha and beta frequency bands (Babiloni et al., 2020; Engels et al., 2015; Engels et al., 2017; Pievani et al., 2011). In predementia AD, however, both increased (theta band) and decreased (alpha and beta band) functional connectivity have been reported (Engels et al., 2017; Pusil et al., 2019). Phase- and amplitude-based connectivity measures may have different sensitivities for changes in specific frequency bands (Briels et al., 2020). Although the mixed local/interregional nature of the JPE_inv_ complicates direct comparison with conventional functional connectivity studies, this study shows that JPE_inv_ can detect abnormal communication between widely distributed brain regions in a predementia AD stage. Furthermore, the magnitude of the JPE_inv_ differences between SCD and MCI subjects is much larger than the effects reported in other functional connectivity studies. Connectivity measures have so far shown inferior biomarker performance when compared with theta band power (Gouw et al., 2017; Gouw et al., 2021; Scheltens et al., 2018).

### Higher Theta PE in MCI

To determine the added value of the JPE_inv_ we compared the results with those obtained from local PE analysis. We found a clear increase in theta band PE in MCI subjects, in almost all brain regions. Several previous studies have suggested that entropy is actually decreased in AD patients (Ando et al., 2021; Gómez & Hornero, 2010; Maturana-Candelas et al., 2019; Shumbayawonda et al., 2020). In agreement with our present study, Maturana-Candelas et al. (2019) have shown that local entropy can increase in the MCI stage, depending on the timescale at which entropy is calculated. Echegoyen et al. (2020) have also shown that the direction of PE changes may depend upon the frequency band. The increase in theta band PE that was observed in MCI could reflect a transient phase of neuronal hyperactivity due to failure of GABAergic inhibitory interneurons in the cortex (Maestú et al., 2021). Previous studies have already suggested a relation between the excitation/inhibition balance and local measures of signal variability/complexity (Waschke et al., 2021). Future model work will have to establish more firmly whether a consistent relation exists between signal irregularity or entropy and the excitation/inhibition balance. A link between micro- and macroscale neurophysiological phenomena in AD is a highly desired translational feature (Maestú et al., 2021) and would strengthen the basis for using entropy measures as biomarkers. The increase in local theta band PE can also be related to the JPE_inv_ findings: Higher local entropy and less interregional coupling will produce *lower* JPE_inv_. By computing both PE and JPE_inv_, it may be possible to determine the relative contribution of local dynamics and interregional connectivity to the early stages of AD.

### The Role of Parameters in Entropy Computations

Computation of entropy measures such as the PE and JPE_inv_ requires the choice of various parameter settings. The pattern size *n* determines the total number of different patterns (*n*!). In the original paper by Bandt and Pompe (2002), it was recommended to choose *n* such that *n*! is much smaller than the length of the time series. This restriction is necessary to obtain reliable statistics for the probability distributions. In the present study we choose a relatively high value (*n* = 4), to compensate for the loss of patterns due to our correction method for the effects of volume conduction/field spread (King et al., 2013). To demonstrate the validity of the selected symbol size, we repeated the JPE_inv_ and PE analysis for different settings of *n* (Supporting Information, Figures 1–3). Another important parameter is time-delay tau (τ). For an illustration of the effect of using different values for tau, see Figure 1D in the present article, or Figure 2 in Kottlarz et al. (2021). In agreement with Bandt and Pompe (2002), we used τ = 1 for our main analysis. To explore the possible effect of another choice for tau, we repeated the analysis of JPE_inv_ and PE for tau = sample frequency/ (3 × high-frequency filter) (Montez et al., 2006); see the Supporting Information, Supplementary Figures 4 and 5. This choice of tau was motivated by the argument that tau should be small enough to capture the highest frequencies present in the signal after filtering. The results for different values of tau indicated a shift of significant group differences from the theta and alpha bands to broadband data. For the JPE_inv_, the direction of the difference between SCD and MCI subjects also changed. The dependency of entropy results on the time delay parameter tau and frequency band is in line with the notion of multiscale entropy as introduced by Costa et al. (2002). According to the multiscale concept, the magnitude and direction of entropy differences depend upon the timescale. This can be explored by creating coarse-grained versions of the original time series and plotting entropy as a function of these timescales. Although this coarse-graining procedure has become very popular in entropy studies, its interpretation is not without problems (Courtiol et al., 2016; Kosciessa et al., 2020). We therefore combined broadband and narrowband filtering with different settings of tau as an alternative procedure. Our results confirm the importance of temporal scale for entropy measures.

### Classification of Individuals

The diagnostic ability of the JPE_inv_ and PE was compared with that of relative theta power, the most potent neurophysiological biomarker of predementia AD to date (Babiloni et al., 2021; Gouw et al., 2017; Gouw et al., 2021; Rossini et al., 2020). We restricted our analysis to mean JPE_inv_, PE, and relative power of data filtered in the theta band. ROC analysis based upon logistic regression showed that theta band JPE_inv_ had the highest AUC. PE and theta power had identical AUC, which was slightly lower than that obtained with JPE_inv_. One-way ANCOVA with relative theta power as a covariate was performed to control for the potential confounding effect of oscillatory slowing on the JPE_inv_ analysis. A significant effect of diagnostic group on mean theta JPE_inv_ was still present after controlling for mean relative theta power, suggesting that the entropy variance between groups can at least partially be explained by nonlinear characteristics. The measure therefore has potential to outperform “pure” theta power as a biomarker for early AD. Since the present study was rather small, and we did not have an independent test set, we cannot yet conclude that the JPE_inv_ performs significantly better than PE or theta power. However, without any extensive optimization, the accuracy obtained with the JPE_inv_ and PE falls within the same range as the current “gold standard” in early AD: relative theta power (Gouw et al., 2017; Gouw et al., 2021; Scheltens et al., 2018). Our results are comparable to those obtained with the multiscale entropy in a study in advanced AD patients (Ando et al., 2021). Further improvement of the JPE_inv_ approach, for instance by optimizing tau and *n*, or by making use of state-of-the-art machine learning techniques, holds promise to deliver a highly sensitive biomarker.

### Limitations

Weaknesses of the present study were its relatively small size and the lack of an independent test set. For artifact removal we relied on our clinical pipeline, which included tSSS (Taulu & Simola, 2006), but we did not use additional tools such as independent component analysis. It should however be noted that the use of extensive preprocessing in combination with entropy analysis has been questioned (Ando et al., 2021). During JPE_inv_ analysis we corrected for volume conduction in a rigorous way, following the proposal by King et al. (2013). While this conservative approach may have caused a certain amount of true functional connectivity to be ignored, we still obtained striking group differences. The influence of volume conduction correction rigidity should be addressed in follow-up studies. Although we did observe significant group differences in JPE_inv_ and PE for frequency bands other than the theta band, we refrained from (over)interpretation of these findings, since much fewer regions (except for alpha band JPE_inv_) were involved and group differences were small. Future studies with larger groups should confirm these findings. We furthermore only used mean values, averaged over all ROIs, in the theta band as input for the classification analysis. Use of a larger range of input features, including information from different ROIs and different frequency bands, in combination with sophisticated machine learning, will probably produce higher sensitivity and specificity values. This was not considered justified in the present study because of its small size and exploratory character.

### Conclusion

To conclude, we have shown that a multivariate version of the permutation entropy holds promise as a biomarker for early-stage Alzheimer’s disease. The method could clearly separate subjects with MCI due to AD from control subjects with subjective cognitive complaints. Preliminary analysis of classification results shows that performance of the JPE_inv_ falls within the same range as relative theta power—currently the most effective neurophysiological biomarker for early AD. Future studies will have to replicate and extend these results in larger samples, preferably with longitudinal data. These samples should include individuals along the entire AD continuum. The present findings could form the starting point for further improvement of complexity-based measures and the use of advanced machine learning techniques. Finally, computational (neural mass) modeling may help to relate measures of neural variability and complexity such as the JPE to underlying neuronal excitation/inhibition (im)balance, in order to gain a better mechanistic understanding of AD.

## ACKNOWLEDGMENTS

The authors would like to thank the participants of the Amsterdam Dementia Cohort for their contribution. Research of Amsterdam Alzheimer Center is part of the neurodegeneration program of Amsterdam Neuroscience. The Amsterdam Alzheimer Center is supported by Alzheimer Nederland and Stichting VUmc funds. The authors thank technicians P. J. Ris, C. H. Plugge, N. Sijsma, N. C. Akemann, N. Zwagerman, and M.C. Alting Siberg for acquisition of the MEG data.

## SUPPORTING INFORMATION

Supporting information for this article is available at https://doi.org/10.1162/netn_a_00224.

## AUTHOR CONTRIBUTIONS

Elliz P. Scheijbeler: Formal analysis; Writing – original draft. Anne M. van Nifterick: Writing – original draft. Cornelis J. Stam: Conceptualization; Formal analysis; Software; Writing – original draft. Arjan Hillebrand: Conceptualization; Writing – review & editing. Alida A. Gouw: Writing – review & editing. Willem de Haan: Data curation; Writing – review & editing.

## TECHNICAL TERMS

Magnetoencephalography (MEG): Noninvasive, high-resolution technique to detect fluctuations in electromagnetic field strength around the head, generated by the active brain.
Volume conduction: Propagation of neural activity through surrounding brain tissue, potentially picked up simultaneously by multiple sensors, complicating description of interregional coupling.
Spectral analysis: Technique to quantitatively describe properties of the oscillatory signal generated by large groups of active neurons, based on frequency and amplitude.
Joint permutation entropy: Measure of both complexity/variability and functional connectivity, based on the temporal similarity in permutation entropy levels of different brain regions.
Nonlinear coupling: Type of dynamic connectivity between elements of a system in which the changes in one element are not proportional to the simultaneous changes in the other element.
Permutation entropy: Measure of complexity/variability, where the entropy is based on the recurrence likelihood of short temporal patterns of, for example, local neuronal activity.
Relative power (spectral density): Quantitative spectral analysis measure that describes the contribution of a specific frequency range to an observed neural oscillatory signal.
Mild cognitive impairment (MCI): Condition in which a person experiences cognitive decline, objectively confirmed by cognitive testing, but without substantial interference in daily activities as seen in dementia.
(Shannon) entropy: Measure of complexity/variability, based on the occurrence likelihood of specific distributions of elements/states in a system (e.g., local neuronal activity levels in a brain network).
Functional connectivity: Large-scale, dynamic neuronal communication between different brain regions, assumed to be estimated by the statistical dependence between elements (e.g., phase) of their signals.
