# Erratum: Network-level permutation entropy of resting-state MEG recordings: A novel biomarker for early-stage Alzheimer’s disease?

**DOI:** 10.1162/netn_x_00380

**Published:** 2024-07-01

**Authors:** Elliz P. Scheijbeler, Anne M. van Nifterick, Cornelis J. Stam, Arjan Hillebrand, Alida A. Gouw, Willem de Haan

After publication of our paper *Network-level permutation entropy of resting-state MEG recordings: A novel biomarker for early-stage Alzheimer’s disease?*, we noticed an error in the correction for degrees of freedom in the statistical analysis of some of the results. This required revision of some of the figures and text throughout the manuscript. In the paper, we performed quantitative analysis on the first 20 epochs of the eyes-closed, resting-state MEG recording of each subject. Relative theta power, permutation entropy, and inverted joint permutation entropy values were estimated for each epoch separately. Inadvertently, the values were not averaged over epochs per person prior to group statistics, inflating the effective degrees of freedom. As a result, the significance of some of the regional results was overestimated. This error was discovered in a re-analysis of the data and is corrected in the revised manuscript.

While the error does not alter the fundamental message of our paper, in particular the classification results reported in Figure 6 and Table 2, it does impact certain reported results. Specifically, the figures and text illustrating regional differences between the SCD and MCI groups. While the direction of effects remains unchanged, the number of regions showing significant group-differences decreases in several instances.

The following is a list of changes made to the main manuscript:

-  Figures 3–5 have been modified to indicate the correct standard error of the mean and significance levels.
-  The Results subsections ‘JPE_inv_’, ‘PE’ and ‘Relative theta power’ were modified to reflect the updated findings.
-  The Discussion subsection previously titled ‘Lower Theta and Alpha JPE_inv_ in MCI’, is now titled ‘Lower Theta JPE_inv_ in MCI’.
-  Discussion subsections ‘Lower Theta JPE_inv_ in MCI’, ‘Higher Theta PE in MCI’, ‘The Role of Parameters in Entropy Computations’ and ‘Limitations’ were modified to describe the updated findings.

The list of changes made to the Supporting Information:

-  Figures S1–S5 have been modified to indicate the correct standard error of the mean and significance levels.
-  Supporting Information sections ‘Embedding dimension: n’ and ‘Time-delay: tau’ were modified to reflect the updated findings.

