# Supplementary material for: Network-level permutation entropy of resting-state MEG recordings: A novel biomarker for early-stage Alzheimer’s disease?
Source: Netw Neurosci. 2022 Jun 1;6(2):382–400. doi: 10.1162/netn_a_00224 (PMC9208018; doi:10.1162/netn_a_00224)
Supplement: Supplementary file 1 [file netn-06-382-s001.pdf]

Scheijbeler, E. P., van Nifterick, A. M., Stam, C. J., Hillebrand, A., Gouw, A. A. & de Haan, W. (2021). Supporting information for "Network-level permutation entropy of resting-state MEG recordings: a novel biomarker for early-stage Alzheimer's disease?" *Network Neuroscience.* Advance publication.
https://doi.org/10.1162/netn_a_00224

Supplementary Table 1. Subdivision of Regions of Interest (ROIs) as included in the automated anatomical labeling atlas. Presented ROI numbers (#) are based upon Gong et al. (2009).

ROI #	Region Name	ROI #	Region Name
**Frontal (L/R)**		**Midline (L/R)**	
1/40	Rectus Gyrus	36/75	Ant. Cingulum
2/41	Olfactory Cortex	37/76	Mid. Cingulum
3/42	Sup. Frontal Orbital	38/77	Post. Cingulum
4/43	Med. Frontal Orbital	39/78	Insula
5/44	Mid. Frontal Orbital		
6/45	Inf. Frontal Orbital	**Occipital (L/R)**	
7/46	Sup. Frontal Gyrus	22/61	Sup. Occipital Gyrus
8/47	Mid. Frontal Gyrus	23/62	Mid. Occipital Gyrus
9/48	Inf. Frontal Opercular	24/63	Inf. Occipital Gyrus
10/49	Inf. Frontal Triangular	25/64	Calcarine fissure
11/50	Med. Sup. Frontal Gyrus	26/65	Cuneus
		27/66	Lingual Gyrus
**Central (L/R)**			
12/51	Supplementary Motor Area	**Temporal (L/R)**	
13/52	Paracentral Lobule	28/67	Fusiform Gyrus
14/53	Precentral Gyrus	29/68	Heschl Gyrus
15/54	Rolandic Operculum	30/69	Sup. Temporal Gyrus
16/55	Postcentral Gyrus	31/70	Mid. Temporal Gyrus
		32/71	Inf. Temporal Gyrus
**Parietal (L/R)**		33/72	Sup. Temporal Pole
17/56	Sup. Parietal Gyrus	34/73	Mid. Temporal Pole
18/57	Inf. Parietal Gyrus	35/74	Parahippocampal Gyrus
19/58	Supramarginal Gyrus		
20/59	Angular Gyrus	**Hippocampus (L/R)**	
21/60	Precuneus	79/80	Hippocampus

The role of parameters in entropy computations

Embedding dimension: n

To obtain reliable statistics for the probability distributions, n! needs to be much smaller than the length of the time-series. In the original permutation entropy paper, Bandt and Pompe (2002) recommend choosing n in the range of 3-7. The value of n determines the number of possible permutations of order n (=n!) as well as the size of the JPE_{inv} probability distribution matrix (=n! * n!). All pairs of identical or mirrored symbols (i.e., the diagonals of the probability distribution matrix) were excluded from computation of the JPE_{inv}. In order to compensate for the loss of patterns due to this volume conduction correction, we selected a relatively high value of n (n=4). To confirm that permutation entropy does not strongly depend on the choice of n, as was previously reported by Bandt and Pompe, we have repeated our PE analysis with the following parameter settings: τ =1 and n=3 & τ =1 and n=5.

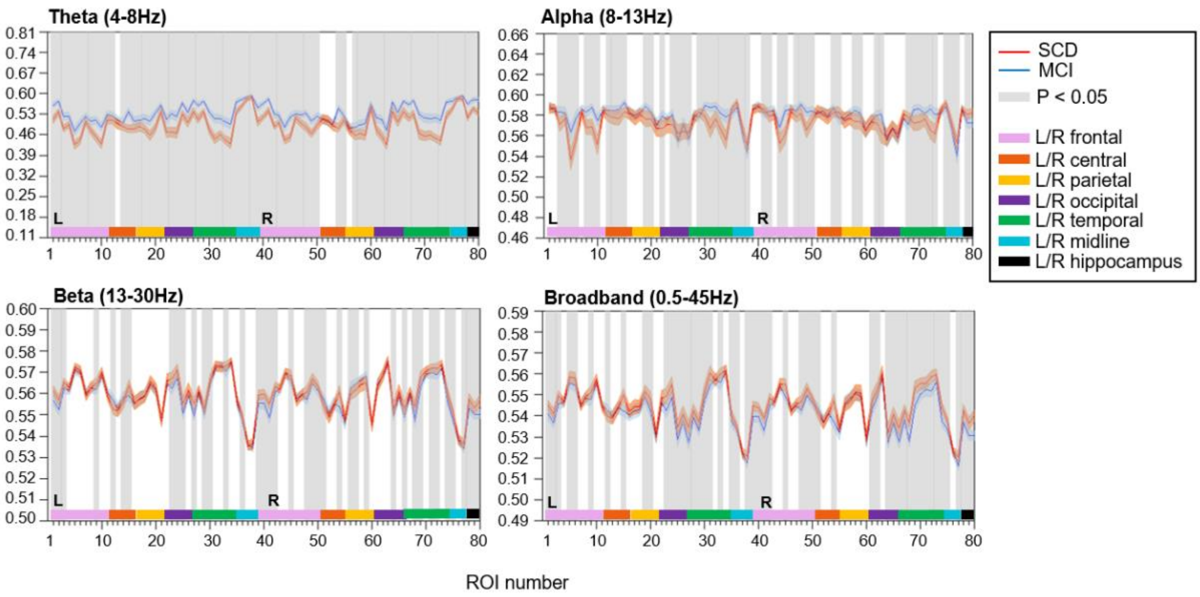

Supplementary Figure 1. Local permutation entropy (τ = 1, n = 3). PE values were calculated for narrowband, i.e. theta (4-8 Hz), alpha (8-13 Hz), beta (13-30 Hz), and broadband (0.5-45 Hz) filtered MEG data. Each ROI number (1-80, order based upon Gong et al. (2009), Supplementary Table 1) represents a brain region in the AAL-atlas. Group means (±2*SEM) are plotted in red for the SCD and in blue for the MCI group. ROIs with significantly different PE values (*q* < .05, FDR corrected) are presented in grey. Group differences were most distinct in the theta band, with 75 channels showing significantly higher PE values for MCI than for SCD subjects. AAL = Automated Anatomical Labeling. FDR = False Discovery Rate. PE = Permutation Entropy. MCI = Mild Cognitive Impairment. SCD = Subjective Cognitive Decline. SEM = Standard Error of the Mean.

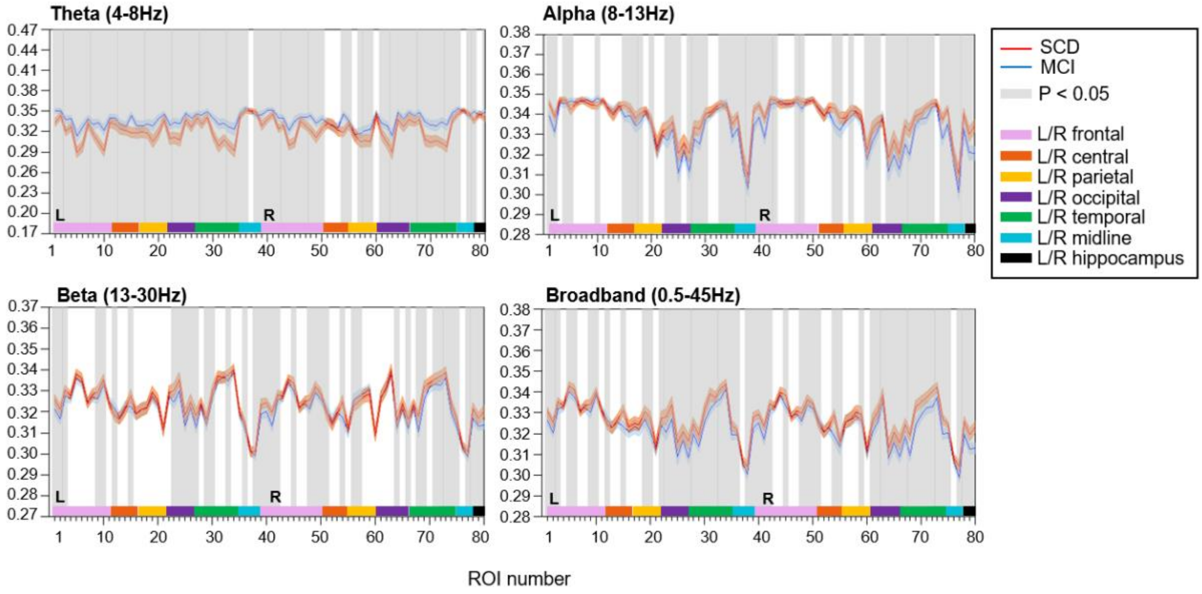

Supplementary Figure 2. Local permutation entropy ($\tau = 1$, n = 5). PE values were calculated for narrowband, i.e. theta (4-8 Hz), alpha (8-13 Hz), beta (13-30 Hz), and broadband (0.5-45 Hz) filtered MEG data. Each ROI number (1-80, order based upon Gong et al. (2009), Supplementary Table 1) represents a brain region in the AAL-atlas. Group means (±2*SEM) are plotted in red for the SCD and in blue for the MCI group. ROIs with significantly different PE values ($q < .05$, FDR corrected) are presented in grey. Group differences were most distinct in the theta band, with 72 channels showing significantly higher PE values for MCI than for SCD subjects. AAL = Automated Anatomical Labeling. FDR = False Discovery Rate. PE = Permutation Entropy. MCI = Mild Cognitive Impairment. SCD = Subjective Cognitive Decline. SEM = Standard Error of the Mean.

Supplementary figures 3 and 4 indicate that shortening (n=3) or lengthening (n=5) of embedding dimension n has little effect on the PE results. While a small shift in absolute PE values can be observed, the embedding dimension does not have a substantial effect on the regional distribution of entropy values, nor on the significant differences between SCD and MCI subjects.

The JPE_{inv} analysis was repeated using parameter settings τ =1 and n=3 (Supplementary Fig. 5). Similar to the PE results, shortening of the embedding dimension had negligible impact on the regional distribution of JPE_{inv} values and the significant group-differences, when compared to the results obtained with τ=1 and n=4 (Fig. 3 in the manuscript). The JPE_{inv} analysis was not repeated for n=5. A pattern size of n=5 would have resulted in a probability distribution matrix of 5! * 5! = 14400 bins / symbol combinations (rather than 4! * 4! = 567 bins). Because the length of the time-series used for analysis was only 4096 samples, a large number of bins in this probability matrix would have remained empty due to a lack of data, rendering computation of the JPE_{inv} unreliable. We believe that these findings demonstrate the validity of the selected symbol size (n=4) for the PE and JPE_{inv} analysis.

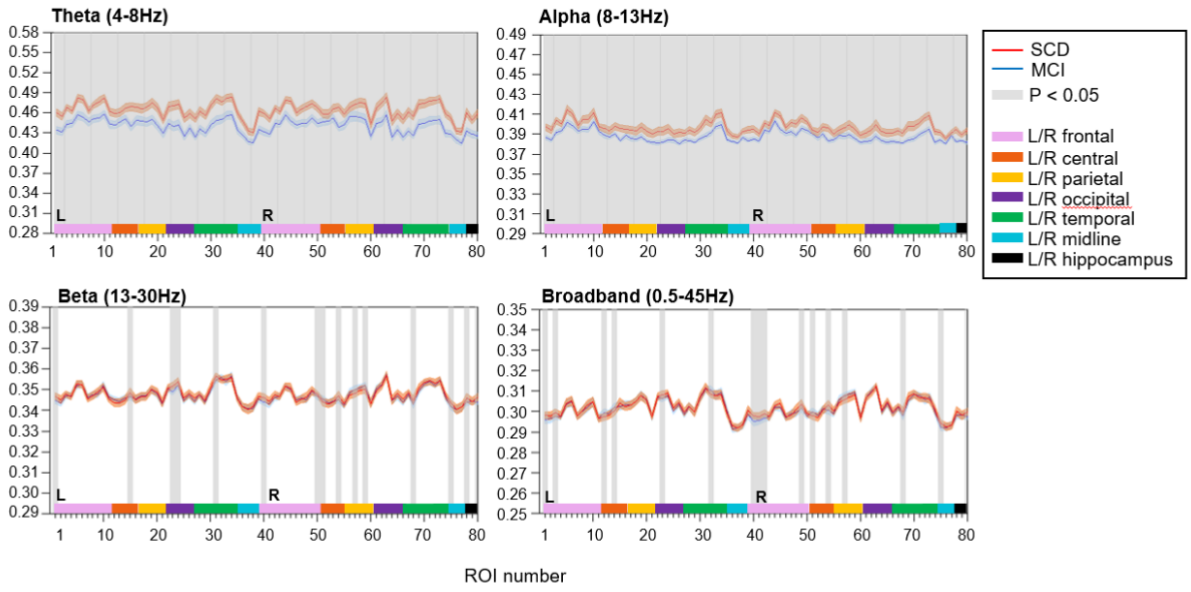

Supplementary Figure 3. Inverted joint permutation entropy (τ = 1, n = 3). JPE_{inv} values were calculated for narrowband, i.e. theta (4-8 Hz), alpha (8-13 Hz), beta (13-30 Hz), and broadband (0.5-45 Hz) filtered MEG data. Each ROI number (1-80, order based upon Gong et al. (2009), Supplementary Table 1) represents a brain region in the AAL-atlas. Group means (±2*SEM) are plotted in red for the SCD and in blue for the MCI group. ROIs with significantly different JPE_{inv} values (q < .05, FDR corrected) are presented in grey. The MCI group presented lower mean JPE_{inv} values for 80 regions in the theta and alpha band. Only a few regions showed significant between-group differences in the beta (i.e., 14) and broadband (i.e., 15) data. AAL = Automated Anatomical Labeling. FDR = False Discovery Rate. JPE_{inv} = Inverted Joint Permutation Entropy. MCI = Mild Cognitive Impairment. SCD = Subjective Cognitive Decline. SEM = Standard Error of the Mean.

Time-delay: tau

To explore the possible effect of another choice for tau we repeated the analysis of JPE_{inv} and PE for tau = sample frequency / (3 * high frequency filter) (Montez et al., 2006). This choice of tau was motivated by the argument that tau should be small enough to capture the highest frequencies present in the signal after filtering. The results for different values of tau indicated a shift of significant group differences from the theta and alpha bands to broadband data. For the JPE_{inv}, the direction of the difference between SCD and MCI subjects also changed.

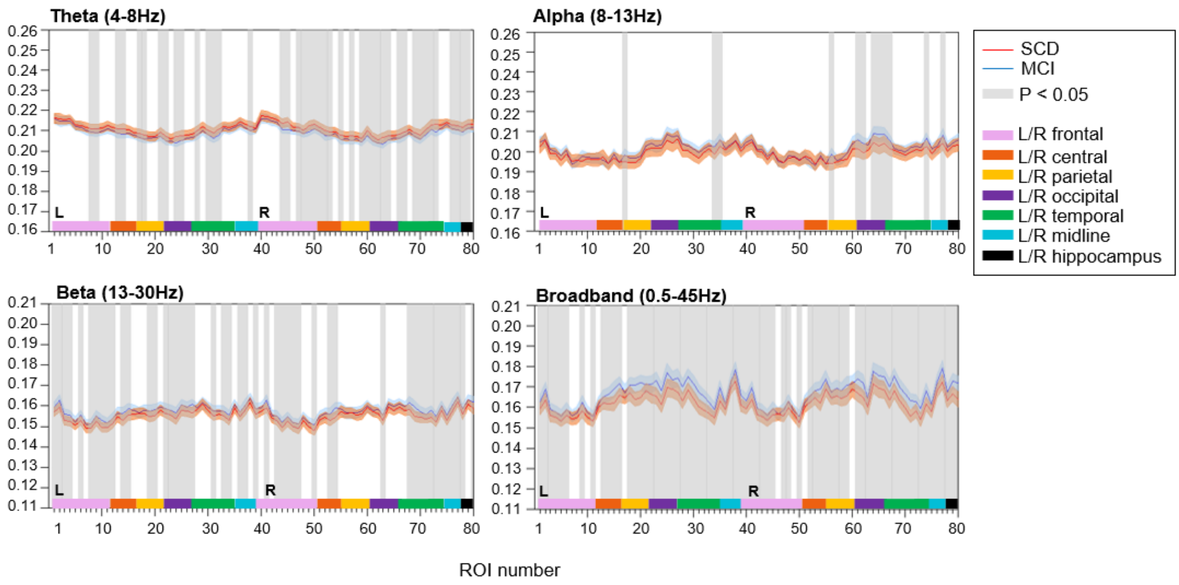

Supplementary Figure 4. Inverted joint permutation entropy (τ = sample frequency / (3 * high frequency filter), n = 4). JPE_{inv} values were calculated for narrowband, i.e., theta (4-8 Hz), alpha (8-13 Hz), beta (13-30 Hz), and broadband (0.5-45 Hz) filtered MEG data. Each ROI number (1-80, order based upon Gong et al. (2009), Supplementary Table 1) represents a brain region in the AAL-atlas. Group means (±2*SEM) are plotted in red for the SCD and in blue for the MCI group. ROIs with significantly different JPE_{inv} values ($q < .05$, FDR corrected) are presented in grey. A clear shift of significant group differences was observed compared to our main analysis. Here, the JPE_{inv} showed most distinct group differences in beta and broadband data, rather than in the theta and alpha frequency bands (Fig. 2: τ = 1, n = 4). AAL = Automated Anatomical Labeling. FDR = False Discovery Rate. JPE_{inv} = Inverted Joint Permutation Entropy. MCI = Mild Cognitive Impairment. SCD = Subjective Cognitive Decline. SEM = Standard Error of the Mean.

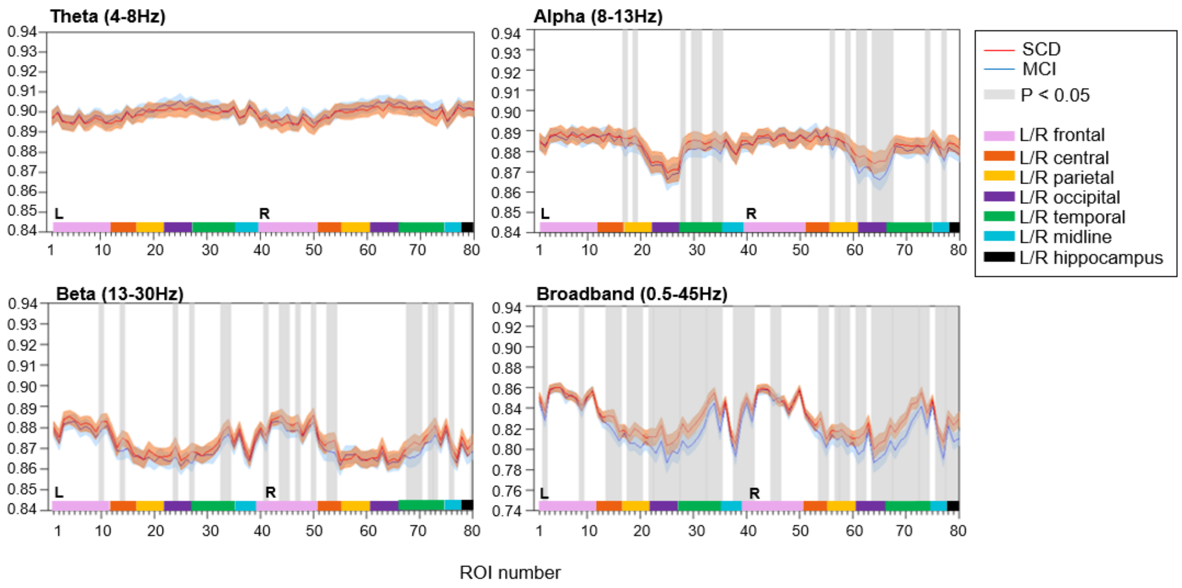

Supplementary Figure 5. Local permutation entropy (τ = sample frequency / (3 * high frequency filter), n = 4). PE values were calculated for narrowband, i.e., theta (4-8 Hz), alpha (8-13 Hz), beta (13-30 Hz), and broadband (0.5-45 Hz) filtered MEG data. Each ROI number (1-80, order based upon Gong et al. (2009), Supplementary Table 1) represents a brain region in the AAL-atlas. Group means (±2*SEM) are plotted in red for the SCD and in blue for the MCI group. Channels with significantly different PE values ($p < .05$, FDR corrected) are presented in grey. In contrast to our main results (Fig. 3: $\tau = 1$, $n = 4$), most significant group differences were found in broadband rather than narrowband filtered data. AAL = Automated Anatomical Labeling. FDR = False Discovery Rate. PE = Permutation Entropy. MCI = Mild Cognitive Impairment. SCD = Subjective Cognitive Decline. SEM = Standard Error of the Mean.
